# Minimizing population health loss due to scarcity in OR capacity: validation of quality of life input

**DOI:** 10.1186/s12874-022-01818-z

**Published:** 2023-01-31

**Authors:** Benjamin Y. Gravesteijn, Kira S. van Hof, Eline Krijkamp, Franck Asselman, C. René Leemans, Anouk M.I.A. van Alphen, Henriëtte van der Horst, Guy Widdershoven, Leonie Baatenburg de Jong, Hester Lingsma, Jan Busschbach, Rob Baatenburg de Jong

**Affiliations:** 1 Department of Otorhinolaryngology, Erasmus University Medical Center, Rotterdam, the Netherlands; 2 Department of Public Health, Erasmus University Medical Center, Rotterdam, the Netherlands; 3 Department of Obstetrics & Gynaecology, OLVG, Amsterdam, Netherlands; 4 Department of Epidemiology, Erasmus University Medical Center, currently employed by the Erasmus School of Health Policy and Management, Erasmus University, Rotterdam, the Netherlands; 5 Strategy & Innovation department, Amsterdam University Medical Centers, Amsterdam, the Netherlands; 6 Department of Otolaryngology – Head and Neck Surgery, Amsterdam University Medical Centres, Cancer Center Amsterdam, Vrije Universiteit, Amsterdam, Netherlands; 7 Department of general practice, Amsterdam University Medical Centers Vrije Universiteit, Amsterdam, Netherlands; 8 Department of Ethics, Law and Humanities, Amsterdam University Medical Centres, Vrije Universiteit, Amsterdam, Netherlands; 9 University of Amsterdam, Amsterdam, Netherlands; 10 Department of Medical Psychology, Erasmus University Medical Center, Rotterdam, the Netherlands

**Keywords:** Validation, Value based health care, Prioritization, Surgery, Decision modeling, Quality of life

## Abstract

**Objectives:**

A previously developed decision model to prioritize surgical procedures in times of scarce surgical capacity used quality of life (QoL) primarily derived from experts in one center. These estimates are key input of the model, and might be more context-dependent than the other input parameters (age, survival). The aim of this study was to validate our model by replicating these QoL estimates.

**Methods:**

The original study estimated QoL of patients in need of commonly performed procedures in live expert-panel meetings. This study replicated this procedure using a web-based Delphi approach in a different hospital. The new QoL scores were compared with the original scores using mixed effects linear regression. The ranking of surgical procedures based on combined QoL values from the validation and original study was compared to the ranking based solely on the original QoL values.

**Results:**

The overall mean difference in QoL estimates between the validation study and the original study was − 0.11 (95% CI:  -0.12 - -0.10). The model output (DALY/month delay) based on QoL data from both studies was similar to the model output based on the original data only: The Spearman’s correlation coefficient between the ranking of all procedures before and after including the new QoL estimates was 0.988.

**Discussion:**

Even though the new QoL estimates were systematically lower than the values from the original study, the ranking for urgency based on health loss per unit of time delay of procedures was consistent. This underscores the robustness and generalizability of the decision model for prioritization of surgical procedures.

**Supplementary Information:**

The online version contains supplementary material available at 10.1186/s12874-022-01818-z.

## Background

Since the beginning of 2020, Covid-19 has put unprecedented pressure on health care worldwide, compromising regular care. The disruptive impact of Covid-19 is noticeable everywhere and has already been described or estimated for orthopedic-, oncological-, HIV- and cardiovascular-healthcare [1–4]. To distribute resources fairly and consistently in times of scarcity such as during a pandemic, a utilitarian perspective has been advocated as most justifiable [5].

To facilitate prioritization of non-acute surgical care, we have previously developed a model that estimates the average expected health loss per unit of time (e.g. months) delay for the most frequently performed semi-elective surgical procedures in our hospital, a university medical center in the Netherlands [6]. We regard this measure, health loss per unit of time delay, as our measure of urgency, since minimizing health loss due to delay results in the highest overall health for the population as a whole. The use of a model that is based on verifiable assumptions and data to guide prioritization has the potential to be more transparent, consistent, and fair compared to prioritization via consensus in discussions with surgical specialists [7]. The current version of the model prioritizes average patients. Therefore, it assists general triage decisions, while leaving room for shared decision making on an individual level. Although this model is developed during the Covid-19 period, it seems also relevant for other situations of scarcity in surgical capacity.

The model comprises seven input parameters describing survival and quality of life of various surgical procedures, which are weighed in the model to estimate the health loss due to delay (see Additional file 1: Table S1 and Table S2). Two of those parameters are the quality of life for the preoperative and postoperative health state. These quality of life values were estimated in focus group meeting of experts from a single university medical center in the Netherlands. Compared to the other parameters which were mostly derived from literature, the estimated quality of life values are more subject to discussion, because expert opinions might be influenced by local circumstances and outcomes. Moreover, they might represent local cultural perspectives on quality of life.

The aim of this study is to validate the ranking of procedures based on urgency as proposed by our model, to overcome the previous limitation of having estimates from only one center. We will test how much the previously proposed ranking is influenced by using estimates from a different university medical center in the Netherlands. If the ranking does not change significantly, we show robustness of our prioritization strategy.

## Methods

### Population

The evaluated surgical procedures in this study comprised non-paediatric, non-obstetric, semi-elective surgical procedures, which are commonly performed in a tertiary university medical center in the Netherlands. From the electronic patient registry (ChipSoft, HiX), data on the frequency of all non-urgent surgical procedures were retrieved from July 2017 to December 2019. Next, two senior clinicians selected the semi-elective surgical procedures from this list. This procedure-level list was rated by both clinicians entirely, and they decided on the final selection in open discussion. A procedure was considered semi-elective if it should ideally be performed within 3 days up to 3 weeks after establishing the indication for surgery. Finally, the expert panel of the original study approved the selection. To focus on the most relevant surgical procedures, we selected surgical procedures that were performed more than 80 times during the retrieval period (an arbitrary value, below which the frequency per procedure declined steeply). Ultimately, 43 semi-elective surgical procedures were selected. The selection consisted primarily of oncological, cardiothoracic, and vascular surgical procedures, and organ transplantations (Additional file 2: Table S1).

### Quality of life values – original study

The quality of life scores of the original study were, where possible, based on the disability weights (quality of life = 1 - disability weight) from the Global Burden of Disease (GBD) study 2016 by the World Health Organization (WHO) [8]. For a total of 34 surgical procedures (out of the 43 selected), we were not able to retrieve the health state from the GBD study. These were therefore estimated in an expert panel meeting in the Erasmus Medical Center (Erasmus MC) in the Netherlands, applying the study protocol of Stouthard et al. [9]. In two meetings, experts were asked to rate the quality of life of the pre- and postoperative health states for all surgical procedures. As a reference, a visual analogue scale (VAS) ranging from 0 (worst imaginable health state) to 100 (best health state imaginable), calibrated by displaying representative GBD health state weights was provided. The experts “mapped” the missing quality of life scores of the 34 surgical procedures onto a calibrated VAS scale (Additional file 1: Fig. S1).

The structure of the discussion was as follows: First, the health state was introduced by the experts most familiar with the patient population. Second, the experts performed an initial anonymous round of scoring. Third, a short plenary discussion of their given weights followed with the aim to reflect on all arguments and to reach a more consensual estimate. After this plenary discussion, everybody gave their final estimate. We deemed the estimates to be internally valid through applying this consensus procedure, and externally valid through calibration to the GBD weights. These sessions were moderated by a medical expert and all sessions were recorded.

### Quality of life values - validation study

To validate the quality of life estimates collected with the Erasmus MC experts, a second panel of medical experts of the Amsterdam UMC was recruited. They were chosen to represent a proportion of surgical and non-surgical specialties similar to the Erasmus MC panel. 18 experts agreed to participate in the study, of which 15 completed both rounds. This time, the quality of life estimates were collected using a web-based Delphi procedure, using the online Delphi software Welphi [10]. The switch from a live meeting to a web-based approach was required due to increasing Covid-19 restrictions.

The recordings of the original expert meetings in the Erasmus MC were transcribed and provided as introductory information to the expert panel from the Amsterdam UMC. Similar to the original study, the experts were asked to anonymously weigh the pre- and postoperative health stage of each condition by mapping the described health state on the calibrated VAS. Additional to giving a weight, the experts had to argue why they gave this weight. In the second Delphi round, the median score and interquartile range of all participants were shown, together with all arguments provided by the participating experts. The weights that the individual expert had given in the first round were also returned to the expert. The experts now had the opportunity to change their initial value based on the provided information of the scoring and arguments of the other experts. In this way, a higher degree of consensus in the final quality of life estimate was reached.

### Statistical analysis

First, the quality of life estimates of the original and validation study were compared. A Bland-Altman analysis was performed to assess systematic differences between estimates in the original and validation study [11]. The Bland-Altman analysis includes calculating 1) the Bland-Altman bias, the systematic error between the validation and the original study, 2) the lower and upper limit of agreement, describing where a new observation is expected to fall when the study is repeated, and 3) the maximum and minimum expected difference in a new validation study. The analysis was visualized in a Bland-Altman plot: On the x-axis, the overall mean of the mean quality of life estimates of the validation and the original study was plotted. On the y-axis, the difference in mean quality of life estimates between the validation versus the original study was plotted. The quality of life estimates of preoperative and postoperative states were analyzed separately. Moreover, to assess the absolute difference between the original and validation study, the standardized mean difference in quality of life estimates between the two studies was calculated by fitting a linear mixed effects model. The dependent variables in this model were all quality of life estimates, and the independent variables were study (validation vs original) and health state (pre- or postoperative). A random intercept was included for procedure. The fixed-effect coefficient for study in this model indicates the standardized difference between the validation and original study estimates.

Second, we assessed the degree of consensus between experts on the quality of life estimates. The standard deviation of the estimates was considered a measure for consensus. A lower standard deviation represents less variability in given scores, which can be interpreted as a higher degree of consensus. We compared these standard deviations between both studies and between surgical procedures. To assess the relationships between the variation, a linear mixed model was fitted using the standard deviations of the quality of life estimates as outcome, the study and pre- and postoperative as fixed effects, and surgical procedure as random intercept. The standardized mean difference between studies was calculated using the coefficient for study, and the variation in consensus was assessed by visualizing the random intercept for procedure. To check model assumptions, we verified whether the residuals of the models were normally distributed around fitted values, as well as with similar variance throughout the range of fitted values (homoscedasticity).

Third, we assessed whether the differences in quality of life estimates between the original and validation study were relevant in terms of the order of DALY/month. This DALY/month is defined as QALY loss per month delay [6]. In order to do so, the decision model estimated the expected health loss due to delay for all surgical procedures again, now using the quality of life estimates of both the original and the validation study. These new estimates of DALY/month delay and consecutively the ranking based on this metric were compared to the results based on the original data only. The rankings were also compared using Spearman’s correlation coefficient.

All analyses were performed in R software [12]. For the linear mixed effects model, the *lmer* function in the *lme4* package was used [13].

## Results

In the original study, 18 experts participated. The validation study panel consisted of 15 experts (table 1). Most experts had surgical specialties: 13/18 [72%] in the original study, and 8/15 [53%] in the validation study.

**Table 1:** Characteristics of the expert panels that participated to get the original data (Erasmus MC) and the validation data (Amsterdam UMC). The panels were from two different University Medical Centers

Specialty	Original data	Validation data
Surgical specialty		
ENT surgeon	1	1
General surgeon	1	–
Neurosurgeon	1	1
Oncological gynaecologist	1	–
Oncological surgeon	2	1
Oncological urologist	–	1
Thoracic surgeon	1	–
Transplantation surgeon	1	1
Trauma surgeon	1	2
Vascular surgeon	2	1
Urologist	2	–
Non-surgical specialty		
Cardiologist	1	2
Final year medical student	1	–
General practitioner	1	1
Geriatrician	2	2
Internist	1	1
Psychologist	–	1
Rheumatologist	1	–
Total	18	15

**Fig. 1:**
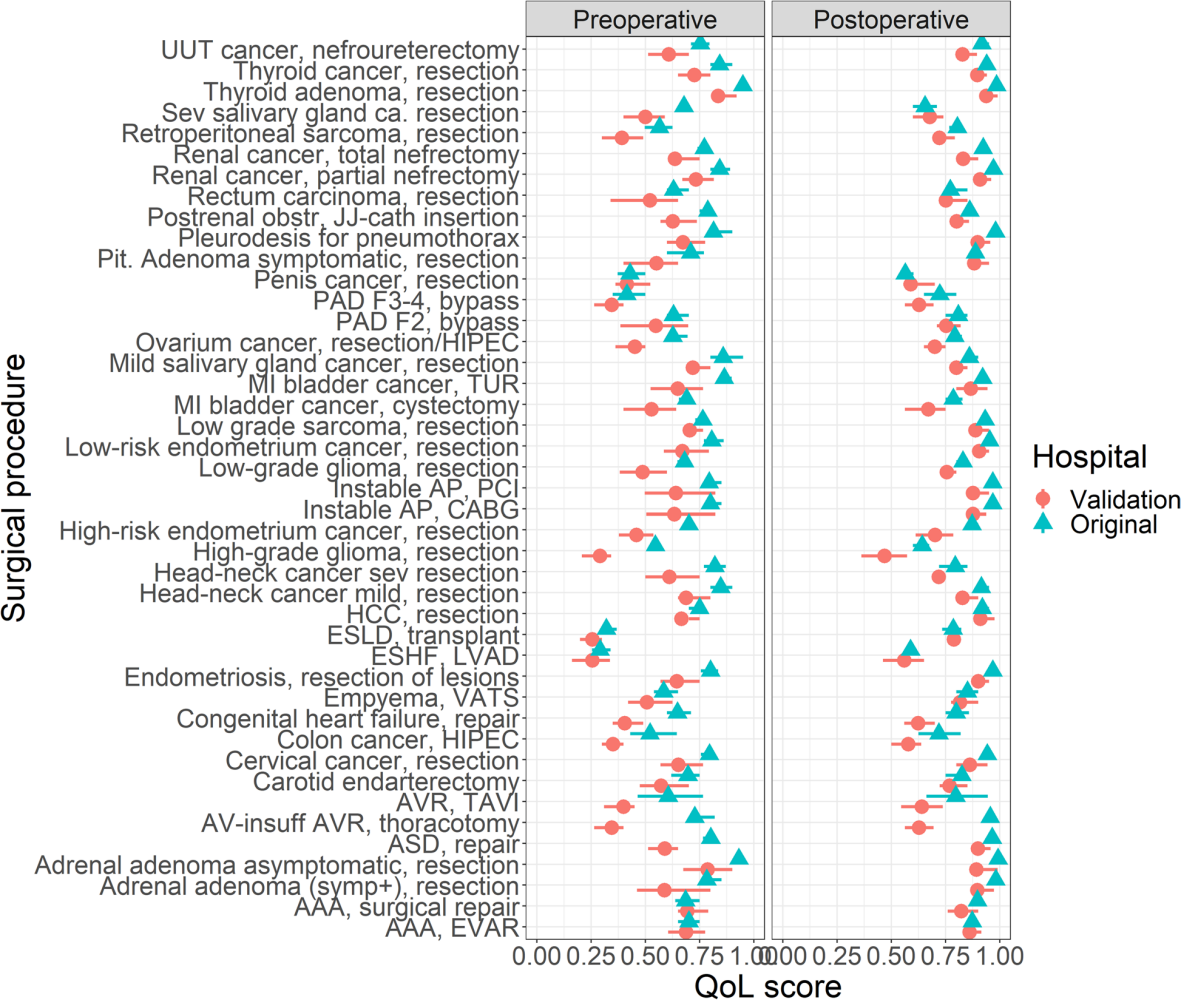
The quality of life estimates derived from the original and the validation study, stratified for preoperative and postoperative state. Abbreviations: AAA, abdominal aneurysm of the aorta; AP, angina pectoris; ASD, atrial septum defect; AV, aortic valve; AVR, aortic valve replacement; CABG, coronary artery bypass graft; COPD, chronic obstructive pulmonary disease; ESHF, end-stage heart failure; ESLD, end-stage liver disease; ESRD, end-stage renal disease; EVAR, endovascular aortic repair; F2, Fontaine 2; F3-4, Fontaine 3-4; HCC, hepatocellular carcinoma; HIPEC, hyperthermic intraperitoneal chemotherapy; LVAD, left ventricle assist device; MI, muscle invasive; NSCLC, non-small cell lung carcinoma; obstr, obstruction; PAD, peripheral arterial disease; PCI, percutaneous coronary intervention; pit, pituitary; sev, severe; TAVI, transcatheter aortic valve implantation; TUR, transurethral resection;UUT, upper urinary tract; VATS, video-assisted thoracoscopy

**Fig. 2:**
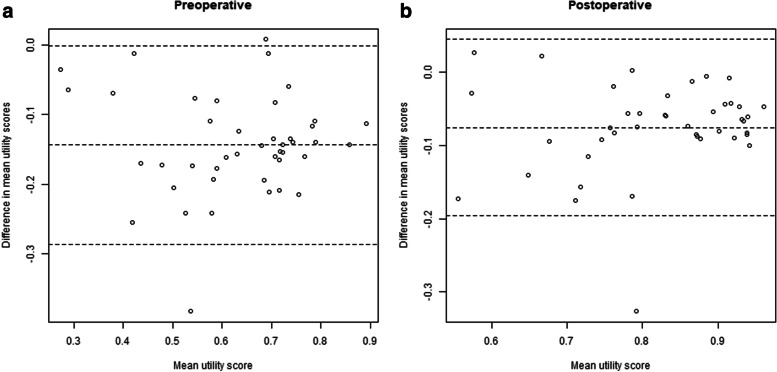
Bland-Altman plot of the orginal and validated quality of life estimates, stratified for the preoperative and postoperative state. The Bland-Altman Bias and 95% limits of agreement are shown (dashed horizontal lines).The mean quality of life estimate on the x-axis represents the overal mean quality of life estimates for the preoperative health state based on data from both the origional study and the validation study. The y-axis represents the difference in mean quality of life estimates between the origninal data and the validation data

**Fig. 3:**
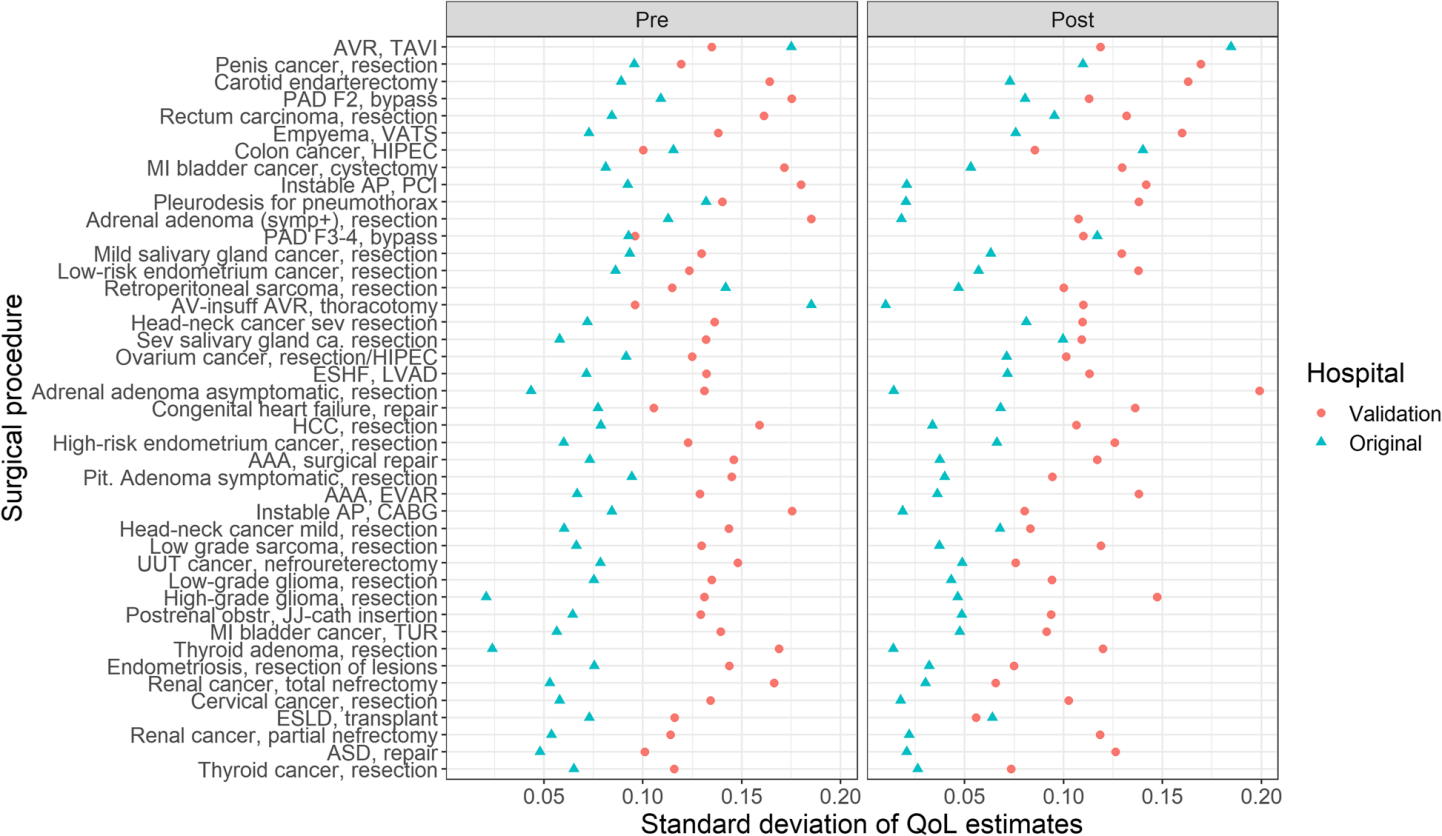
Standard deviation of Quality of life (QoL) estimates for the different surgical procedures and hospitals. Abbreviations: AAA, abdominal aneurysm of the aorta; AP, angina pectoris; ASD, atrial septum defect; AV, aortic valve; AVR, aortic valve replacement; CABG, coronary artery bypass graft; COPD, chronic obstructive pulmonary disease; ESHF, end-stage heart failure; ESLD, end-stage liver disease; ESRD, end-stage renal disease; EVAR, endovascular aortic repair; F2, Fontaine 2; F3-4, Fontaine 3-4; HCC, hepatocellular carcinoma; HIPEC, hyperthermic intraperitoneal chemotherapy; LVAD, left ventricle assist device; MI, muscle invasive; NSCLC, non-small cell lung carcinoma; PAD, peripheral arterial disease; PCI, percutaneous coronary intervention; QoL, quality of live, TAVI, transcatheter aortic valve implantation; UUT, upper urinary tract; VATS, video-assisted thoracoscopy

The pre- and postoperative quality of life estimates of the validation study were systematically lower than those in the original study (Fig. 1 and 2). The Bland-Altman mean bias and lower and upper levels of agreement was -0.14 (-0.29 − 0.00) for the preoperative scores, and -0.08 (-0.20 − 0.04) for the postoperative scores. The overall mean difference in quality of life estimates of the validation study versus the original study was -0.11 (95% CI: 0.12 − -0.10, Additional file 3: Table S1). Furthermore, the consensus between quality of life estimates in the validation study was systematically lower: the standard deviations of the surgical procedures were consistently higher in the validation study (standardized mean difference of 0.06, 95% CI: 0.05 − 0.07; Fig. 3 and Additional file 3: Table S2). The highest degree of consensus was achieved for resection of thyroid cancer, and the lowest degree of consensus was achieved for transcatheter aortic valve replacement, but the variation in degree of consensus over all surgical procedures was low (standard deviation of 0.005, on a scale of 0 to 100, see Additional file 3: Fig. S3).

**Fig. 4:**
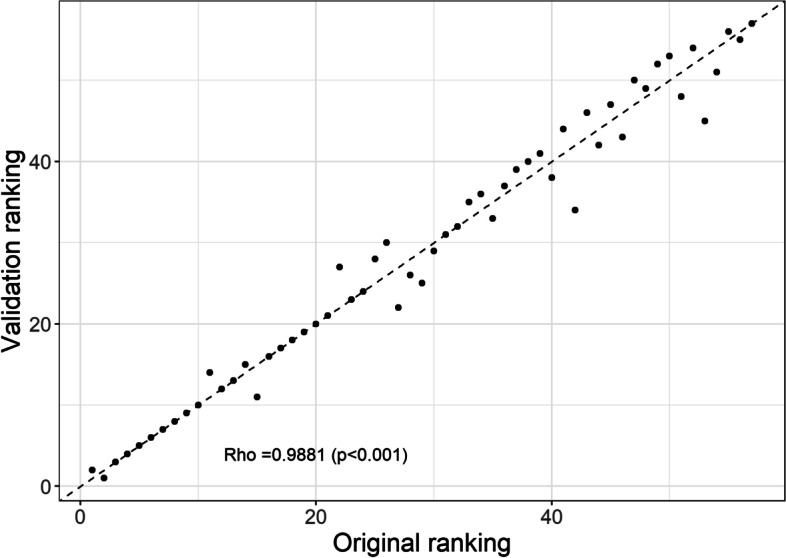
Comparing of the ranking of the procedure based on the original quality of life estimates (x-axis), versus the ranking based on the original and validation study scores (y-axis). Rho is the Spearman correlation coefficient between the original and validation study

**Fig. 5:**
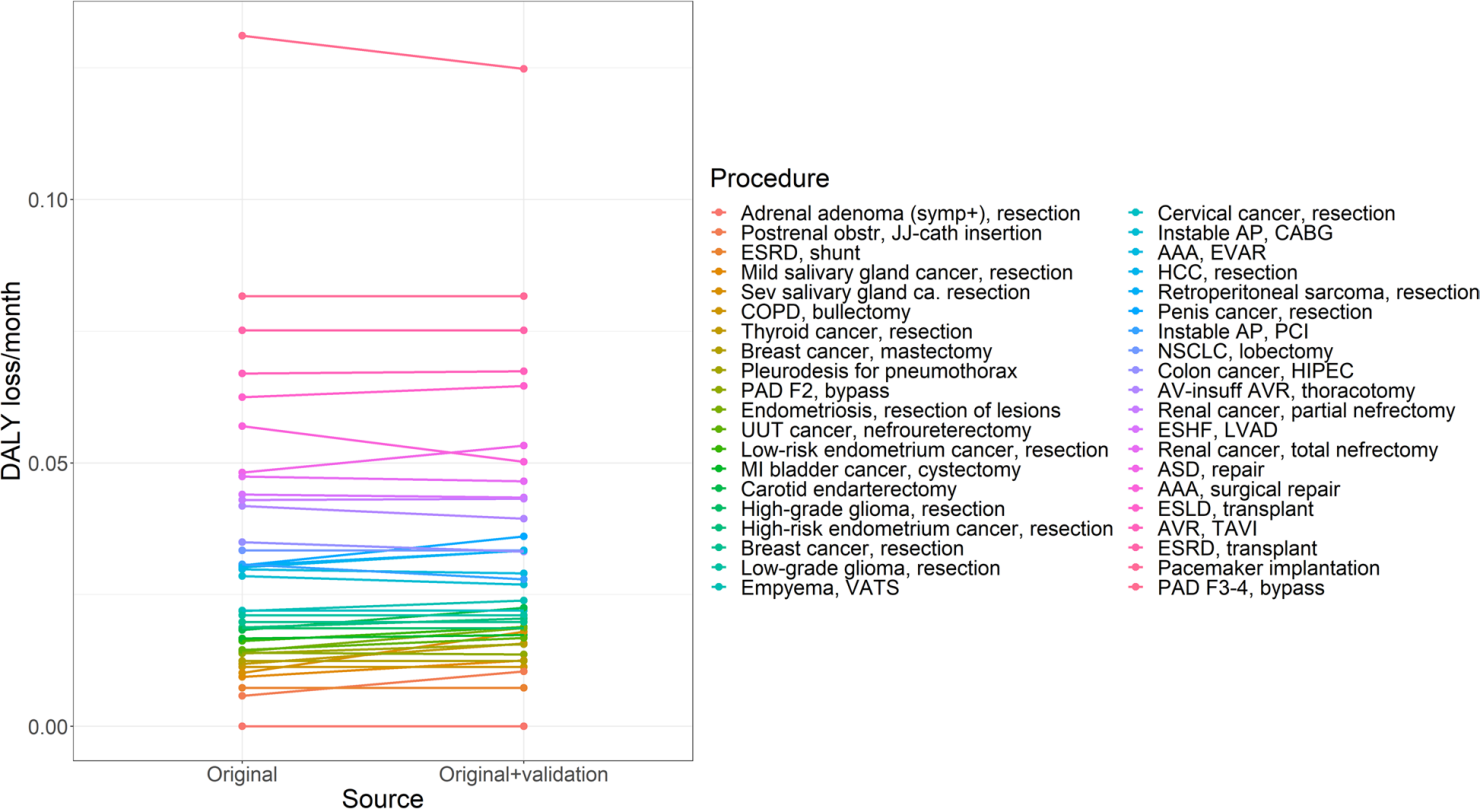
The difference in urgency of surgical procedures between the original and the updated quality of life estimates. Only the diseases which now include the new quality of life estimates from the validation study are shown. Abbreviations: AAA, abdominal aneurysm of the aorta; AP, angina pectoris; ASD, atrial septum defect; AV, aortic valve; AVR, aortic valve replacement; CABG, coronary artery bypass graft; COPD, chronic obstructive pulmonary disease; ESHF, end-stage heart failure; ESLD, end-stage liver disease; ESRD, end-stage renal disease; EVAR, endovascular aortic repair; F2, Fontaine 2; F3-4, Fontaine 3-4; HCC, hepatocellular carcinoma; HIPEC, hyperthermic intraperitoneal chemotherapy; LVAD, left ventricle assist device; MI, muscle invasive; NSCLC, non-small cell lung carcinoma; PAD, peripheral arterial disease; PCI, percutaneous coronary intervention; TAVI, transcatheter aortic valve implantation; UUT, upper urinary tract; VATS, video-assisted thoracoscopy

This systematically lower quality of life estimates for health states did not translate into a substantially different ranking of the various health conditions. The Spearman correlation coefficient between the two different rankings was 0.99 (*p* < 0.001, Fig. 4, and Additional file 3: Fig. S2). Including the new quality of life estimates also did not result in substantially different estimates for DALY/month delay (Fig. 5). The largest difference in urgency between results based on the updated data (original + validation) and based on original data was found for surgical repair of aneurysm of the abdominal aorta. The health loss associated with postponing this procedure was 0.06 (0.05-0.07) DALY/month delay in the original study, and 0.05 (0.04 – 0.06) DALY/month delay in the validation study. This effectively resulted in a lower ranking of surgical repair of the abdominal aorta: it dropped from 11th to 14th place.

## Discussion

This study aimed to replicate the estimation of the quality of life values for various healthy states used in the model we developed in our previously published study, and to validate the output of the decision model with updated quality of life estimates. We found that the quality of life estimates were systematically lower in the validation study compared to the original study. Moreover, the degree of consensus reached in the validation study was lower than in the original study. Nevertheless, this systematic difference did not substantially impact the estimates for health loss per month in case of delayed surgery. More importantly, the ranking based on these updated estimates was found to be consistent with the original study.

The observation of systematically lower quality of life estimates at validation might have been caused by differences in the methodology between the original and the validation study. The systematic lower degree of consensus between experts is another observation which supports this hypothesis. Whereas the original study was performed as in-person focus group, the current validation study was a web-based Delphi approach [10, 14]. Data richness, in terms of unique ideas and argumentative depth, is highest for in-person focus groups [15, 16]. Nevertheless, the structure that the web-based Delphi brings compared to an online focus group increases data richness [16] and is therefore preferred over online focus groups when in-person focus groups are not feasible. The relation between the web-based Delphi procedure and the lower estimated quality of life estimates is not completely clear. Some specific differences that we believe might have increased the difference between the two methods are 1) that in the original study, patients were introduced by the specialist that primarily cares for these patients, while in the validation study, patients were introduced by a text description; 2) that in the validation study, all input from the discussion in the original study (extra symptoms, patient experience) was already given in the first description; 3) that in the validation study, the proportion of surgical specialists was lower (despite our efforts of putting together a similar panel); 4) that in the validation study, the provided comments were anonymous, while in the original study, the specialism of all participants was known; 5) that in the original study, the average score of the first guess was not known to participants before they gave their final answer, while in the validation study, the participants knew what their initial score was compared to the average of the primary, non-final answers.

Although there is a systematic difference in quality of life estimates between the validation and original study, the impact on the final results of the model is not substantial as the ranking based on the DALY per month delay of surgery is consistent. This finding is in line with observations in other DALY/QALY research: where survival and quality of life interact, usually the quality of life component has only a limited influence compared to survival [17, 18]. Also, the difference in quality of life score seem to be relatively constant over the health states. As a consequence, the ordering of the procedures on the basis of other quality of life scores is relatively constant. Moreover, the difference between quality of life scores of a health state with or without waiting is not affected if both the health state with or without waiting are move over scale with a constant. All this makes that, although it is tempting to think that the results of the model are influenced by the estimation of quality of life, it turns out that any influence of possible difference in the quality of life estimation is limited. Rather, as also observed in the original paper [6], the contribution of survival to prioritization is higher than the contribution of quality of life.

## Limitations

The most important limitation of our study is that we used a different methodology to obtain quality of life estimates in the validation study, compared to the original study. As discussed above, this might have led to systematically different quality of life estimates. Nevertheless, the most relevant output (the ranking of surgical procedures) was similar between the two studies.

Another limitation of our study is that we validated the quality of life estimates only in one other academic hospital. Still, this exercise did show empirical evidence that the prioritization strategy is insensitive to small differences in quality of life estimates. The generalizability to other academic hospitals is therefore very likely.

Also, we did not include patient representatives in this validation study, similar to the original study. We think that a more distanced perspective (experts opinion) on quality of life is more helpful in triage decisions, as patient reported evaluation of health may be biased due to distortion by coping mechanisms. However, a future study will focus on including a citizen perspective.

Moreover, both the original and the validation study did not include a very large group of experts. Lower sample sizes might also result in more extreme, less certain estimates. Nevertheless, the variance in both studies was not substantial, and consequently the confidence intervals of the quality of life estimates are narrow enough for the model to translate into a relevant discriminatory ranking. Moreover, our sample size is within normal range of other Delphi panels (e.g.: 6-50 participants [19]).

## Implications

This validation study has empirically assessed the robustness of our prioritization strategy against the quality of life estimates. The ranking was insensitive to a systematic difference in quality of life estimates. In order to improve decision making, we recommend focusing on the validity in the survival parameters in the further development of this prioritization strategy. These parameters should therefore repeatedly be updated when newer, higher quality evidence is available, since these apparently drive the utilitarian prioritization strategy.

Although our results replicate towards other academic hospitals in the Netherlands, we think that the results also replicate towards other settings where these set of procedures are performed. A larger obstacle towards applying our model in other parts of the world, is the inclusion of a finite panel of procedures. We need to include more procedures and input parameters applicable to a specific setting. This would also facilitate the transfer of our prioritization strategy towards parts where triage is especially critical, for example in low-middle income countries (LMIC). However, it is crucial to adjust input parameters according to those settings.

Moreover, another necessary developmental step is towards implementation. The first critical step we now see for implementation is to develop a strategy to optimally distribute hospital capacity according to urgency of the surgical procedures. This strategy should also take co-morbidity and individual context into account.

## Conclusion

A systematic difference in quality of life estimates was seen between the validation and the original study, but this systematic difference in quality of life estimates did not translate into seriously different estimates for health loss (DALY) per month delay of surgery. This study underscores the robustness and generalizability of the decision model for prioritization of surgical procedures based on DALY loss per month delay. The ranking based on our prioritization strategy is consistent, and now more likely transportable to settings where a similar set of procedures are performed.

## Supplementary Information

**Additional file 1:** **Fig. S1.** Calibrated visual analog scale based on the Global burden of disease study. **Table S1.** Description of input parameters. **Table S2.** Form for participants in the Delphi rounds in the developmental study.

**Additional file 2: Table S1.** Descriptions in the Delphi round to introduce the procedures.

**Additional file 3: Fig. S1.** The structure of the previously developed cohort state-transition model. Preop: preoperative state; Postop: postoperative state (6). **Fig. S2.** The model estimates for urgency based on the original quality of life estimates (upper panel) and the updated scores from both the original and the validation study (bottom panel). **Fig. S3.** The random effects of procedure on the standard deviation of the QoL estimates. These estimates are the random intercept values for procedure in a model with as independent variable the standard deviations of surgical procedures, also including hospital and pre- or postoperative as fixed effects (supplementary table 2). A random intercept above 0 indicates a higher than expected standard deviation, which we interpret as lower consensus between experts. A random intercept below 0 indicates a lower than expected standard deviation, which we interpret as higher consensus between experts. The overall standard deviation of the random effect was 0.005. **Table S1.** The estimates from the first mixed effects linear regression model. The dependent variable is the utility scores scored by the expert panel. **Table S2**. The estimates from the second mixed effects linear regression model. The dependent variable is the standard deviation of the utility scores per study center, pre- and postoperative state, and procedure. **Table S3.** The quality of life estimates and 95% CI derived from the original study and the validation study, stratified for preoperative and postoperative state, corresponding to figure 1 in the manuscript. **Table S4.** The difference in urgency of surgical procedures between the original and the updated quality of life estimates. Only the diseases which now include the new scores from the validation study are shown. This table corresponds to figure 4 in the manuscript.

## Data Availability

The datasets generated and/or analysed during the current study are not publicly available due to the nature of sensitivity of professional opinions, but will be made available anonymized by the corresponding author on reasonable request.
