# Supplementary material for: Minimizing population health loss due to scarcity in OR capacity: validation of quality of life input
Source: BMC Med Res Methodol. 2023 Jan 31;23:31. doi: 10.1186/s12874-022-01818-z (PMC9887555; doi:10.1186/s12874-022-01818-z)
Supplement: Supplementary file 1 — Additional file 1: Fig. S1. Calibrated visual analog scale based on the Global burden of disease study. Table S1. Description of input parameters. Table S2. Form for participants in the Delphi rounds in the developmental study. [file 12874_2022_1818_MOESM1_ESM.zip › Additional file 1_Table S1.docx]

Additional file 1: Table S1 Description of input parameters

| Parameter | Description |
| --- | --- |
| Age | Average age of the patient population. |
| Preoperative survival rate | The survival rate of patient before they underwent the surgical procedure. |
| Postoperative survival rate | The survival rate of patient after they underwent the surgical procedure. |
| Preoperative quality of life | The quality of life of patient before they underwent the surgical procedure. |
| Postoperative quality of life | The quality of life of patient after they underwent the surgical procedure. |
| Time until no effect on quality of life | The time until no effect from the procedure can be expected on quality of life. |
| Time until no effect on survival | The time until no effect from the procedure can be expected on survival. |

Supplementary figure 1, Calibrated visual analog scale based on the Global burden of disease study. Data source: IHME_GBD_2016_DISABILITY_WEIGHTS_Y2017M09D14 (downloaded may 2020 from http://ghdx.healthdata.org/gbd-2016) More about this data can be found here: <http://www.healthdata.org/gbd/about/history>

Supplementary table 4, form for participants in the Delphi rounds in the developmental study.

| Name expert: |  |
| --- | --- |
| Target population/disease: |  |
| Preoperative | Postoperative |
| Stage 1-2/mild/moderate | Stage 1-2/mild/moderate |
|  |  |

Supplementary table 4, continued.

| Name expert: |  |
| --- | --- |
| Target population/disease: |  |
| Preoperative | Postoperative |
| Stage 3-4/severe | Stage 3-4/severe |
|  |  |
