# Supplementary material for: Minimizing population health loss due to scarcity in OR capacity: validation of quality of life input
Source: BMC Med Res Methodol. 2023 Jan 31;23:31. doi: 10.1186/s12874-022-01818-z (PMC9887555; doi:10.1186/s12874-022-01818-z)
Supplement: Supplementary file 1 — Additional file 1: Fig. S1. Calibrated visual analog scale based on the Global burden of disease study. Table S1. Description of input parameters. Table S2. Form for participants in the Delphi rounds in the developmental study. [file 12874_2022_1818_MOESM1_ESM.zip › Additional file 1_Table S2.docx]

Additional file 1: Table S2 Form for participants in the Delphi rounds in the developmental study.

| Name expert: |  |
| --- | --- |
| Target population/disease: |  |
| Preoperative | Postoperative |
| Stage 1-2/mild/moderate | Stage 1-2/mild/moderate |
|  |  |

Supplementary table 4, continued.

| Name expert: |  |
| --- | --- |
| Target population/disease: |  |
| Preoperative | Postoperative |
| Stage 3-4/severe | Stage 3-4/severe |
|  |  |
