# Supplementary material for: Minimizing population health loss due to scarcity in OR capacity: validation of quality of life input
Source: BMC Med Res Methodol. 2023 Jan 31;23:31. doi: 10.1186/s12874-022-01818-z (PMC9887555; doi:10.1186/s12874-022-01818-z)
Supplement: Supplementary file 2 — Additional file 2: Table S1. Descriptions in the Delphi round to introduce the procedures. [file 12874_2022_1818_MOESM2_ESM.zip › Additional file 2_Table S1.docx]

Additional file 2: Table S1 Descriptions in the Delphi round to introduce the procedures.

| Procedure | Description in Delphi |
| --- | --- |
| AAA, EVAR | Endovascular aortic repair for abdominal or iliacal aneurysms.  **Preoperatively**, patients are asymptomatic. The aneurysm is found per accident, or during screening. The disease does not impact quality of life by limiting physical wellbeing, but the patient is aware of the aneurysm within. This awareness can be tough for patients, impacting mental wellbeing.  **Postoperatively**, these patients have a high risk on complications, for which they need multiple extra procedures. Most common complication is prothesis endoleaks, for which new endovascular procedures can fix the leak. We assume that these complications do not occur for the estimation of quality of life. However, the awareness of the possibility of these complications might impact mental wellbeing. |
| AAA, surgical repair | Transabdominal aortic repair for abdominal aneurysms.  **Preoperatively**, patients are asymptomatic. The aneurysm is found per accident, or during screening. The disease does not impact quality of life by limiting physical wellbeing, but the patient is aware of the aneurysm within. This awareness can be tough for patients, impacting mental wellbeing.  **Postoperatively**, compared to the EVAR procedure, the transabdominal procedure has more risk on perioperative morbidity, but lower risk on complications. |
| Adrenal adenoma asymptomatic, resection | Laparoscopic resection of an asymptomatic adrenal adenoma.  **Preoperatively**, these patients are asymptomatic. Often, these adenomas are found by accident. Patients can have decreased mental wellbeing due to the insecurity about this neoplasm.  **Postoperatively**, these patients have almost no physical limitations. |
| Adrenal adenoma symptomatic, resection | Laparoscopic resection of a symptomatic adrenal adenoma.  **Preoperatively**, these patients can have severe untreated hypertension, caused by the adrenal adenoma. Some patients are known to suffer from hypertension and/or migraines. Moreover, patients can have symptoms of restlessness.  **Postoperatively**, these patients have almost no physical limitations. |
| ASD, repair | Repair of a congenital atrial septum defect.  **Preoperatively**, these patients are young and active, in the middle f their life. Because of their condition, they have trouble keeping up with their peers. The symptoms are more severe than patients with for example an aorta valve stenosis, because they have a more active lifestyle (or at least, want to have).  **Postoperatively**, these patients are no longer held back by their condition. |
| AV-insuff AVR, thoracotomy | Thoracotomy for aortic valve insufficiency.  **Preoperatively**, due to the symptomatic valve insufficiency, these patients suffer from dyspnea and decreased condition. These patients have high NYHA-classifications, and have a high age of 65-70 years. However, some patients with bicuspid aortic valves also have this disease. The quality of life is substantially decreased due to the symptoms.  **Postoperatively**, these patients have increased quality of life. If they look back on their preoperative condition, they are aware of how much they have suffered. |
| AVR, TAVI | Transcatheter aortic valve replacement.  **Preoperatively**, due to the symptomatic valve insufficiency, these patients suffer from dyspnea and decreased condition. These patients have high NYHA-classifications, and have a high age of 65-70 years. However, some patients with bicuspid aortic valves also have this disease. The quality of life is substantially decreased due to the symptoms.  **Postoperatively**, these patients have increased quality of life. If they look back on their preoperative condition, they are aware of how much they have suffered. |
| Carotid endarteriectomy | Endarterectomy of symptomatic stenosis of the carotid artery.  **Preoperatively**, these patients have worsened vascular condition. Due to atherosclerosis in the carotid artery, small emboli are being shot towards the cerebral vasculature. Sometimes, these cause a transient ischemic attack (TIA), but sometimes, a stroke with long lasting damage follows. The patient group eligible for surgery often have had multiple strokes or TIA’s. Where possible, they have already recovered from these. If a stenosis is found in the work-up after recovery, the risk for a new ischemic event is increased. Patients therefor also suffer from the idea that they can get a new stroke.  **Postoperatively**, patients do not feel better, but worsening is prevented. The surgery reduces fear for a new stroke, and increases survival. |
| Cervical cancer, resection | Radical hysterectomy for cervical cancer.  **Preoperatively**, these women are 30-44 years old, with almost no symptoms. They mainly suffer from the idea that they have a potentially lethal disease.  **Postoperatively**, these patients can suffer from urine incontinence due to problems in the innervation of the bladder. Moreover, they lose their uterus, and therefore cannot are sterilized. |
| Colon cancer, HIPEC | Hyperthermic intraperitoneal chemotherapy for peritonitis carcinomatosa caused by colon carcinoma.  **Preoperatively**, these patients have metastasized disease. They have a colon carcinoma, and ascites. Another possibility is that they have had a resection for their colon carcinoma, and in the follow up, ascites accumulates based on peritoneal metastasis. They have an enlarged belly, and possibly pain. Some patients have pseudo-obstruction, and are obstipated. The procedure is in essence curative, because it entails a primary colon carcinoma. However, this procedure is major surgery, which can only be performed when the patient is in the right condition.  **Postoperatively**, patients are cured, but the recovery lasts for a long period. The recurrence rate, unfortunately is high, and leads to an incurable disease. |
| COPD, Bullectomy | Bullectomy for chronic Obstructive Pulmonary Disease with substantial emphysema.  **Preoperatively**, patients are end-stage COPD patients, with substantial symptoms despite maximal pharmaceutical interventions. They are continuously dependent on oxygen, and therefore restricted in their movements.  **Postoperatively**, patients remain severely ill, with often still end-stage COPD. The disease will not change very substantially. However, because of the interventions, they have less risk on complications and infections. Moreover, their condition often does improve after surgery, but not substantially. |
| Congenital heart failure, repair | Surgically repairing a aongenital heart defect, e.g. Fallot’s syndrome.  **Preoperatively**, these patients are mostly young, hospitalized throughout their youth. They are often symptomatic, and have severe functional problems.  **Postoperatively**, these patients do recover, with better functionality than before. However, there are numerous complications to expect. Moreover, they remain a lifelong patient. The quality of life is limited, because they know that they might require more surgery later on. Moreover, they have a higher risk on arrythmias. |
| Empyema, VATS | Video assisted thoracoscopical drainage of spontaneous empyema.  **Preoperatively**, patients are ill and often hospitalized with episodes of fever. Therefore, they have to be drained multiple times. If the empyema lasts long enough, lung capacity decreases irreversibly.  **Postoperatively**, patients are less frequently hospitalized. The episodes of fever and illness are over. After surgery, patients are generally cured, but some will have long lasting lung damage. |
| Endometriosis, resection | Hysterectomy for endometriose  **Preoperatively**, these patients endure monthly episodes of disproportionally painful periods. This pain can be decreased by suppressing their normal cycle. However, for patients requiring surgery, this pharmaceutical intervention is not enough.  **Postoperatively**, these patients experience a substantial improvement in symptoms. They suffer less from their heavy periods, and can function better through these episodes. |
| ESHF, LVAD | Implantation of a left-ventricle assist device for end-stage heart failure.  **Preoperatively**, these patients are severely ill. They are not mobile, and they have severe functional problems. Most often, these patients have had progressively worse problems, where they have had pharmaceutical interventions for years. Often, they are on the waiting list for a heart transplant, which is not yet available. This device is often used to bridge the period between heart failure and heart transplant.  **Postoperatively**, these patients can recover immensely: they are mobile and can take up their daily life (with some basic rules). However, they are stuck with a device that needs to be charged by a battery. They need a partner who is able and willing to assist in the maintenance. |
| ESLD, transplant | Liver transplantation for end-stage liver disease.  **Preoperatively**, patients are weak, nauseous and ill. They are often chronically tired. They have ascites and a poor condition. They are often admitted. Liver transplantation is the only available definitive treatment, which is a large operation.  **Postoperatively**, patients have a functioning liver where they can live with. In terms of functioning, they are almost at the level before severe liver disease. However, they are bound to use immunosuppressive drugs for the rest of their lives, with substantial risk on adverse events (skin malignancies, and lymphomas). These complications however only occur in a minor proportion of patients, and patients do not fear them in general. |
| HCC, resection | Surgical resection of hepatocellular carcinoma  **Preoperatively**, patients have few symptoms and on average a good quality of life. The disease is often found per accident. They do suffer from the knowledge that they have a potentially lethal disease.  **Postoperatively**, patients recover adequately, often without lasting symptoms. They are cured in general. |
| Head-neck cancer mild, resection | Resection of malignous lymph node metastases, often from HPV induced squamous cell carcinoma in the oropharynx, mild.  **Preoperatively**, patients often have no symptoms and present with asymptomatic swollen lymph node in the neck.  **Postoperatively**, the patient have not suffered from the surgery itself. They can suffer from sequelae of the radiotherapy, such as less productive saliva glands. Prognosis is good, in general. |
| Head-neck cancer sev resection | Resection of malignous lymph node metastases, often from squamous cell carcinoma in the oropharynx, severe.  **Preoperatively**, these patients have the same symptoms as the mild version of the disease. This however is a HPV-negative primary tumour, caused by smoking and alcohol. Often, the primary tumor is not found. These patients receive postoperative radiotherapy more often than the mild counterpart.  **Postoperatively**, patients recover worse than the mild version. The prognosis is worse. |
| High-grade glioma, resection | Surgical resection of a high-grade glioma.  **Preoperatively**, these patients have, compared to low-grade glioma, much more severe symptoms. These tumours grow rapidly, causing the symptoms. They often suffer from paralysis of extremities, aphasia. Moreover, they can suffer from intense headaches and nausea and vomiting. If they do not receive surgery, they can die within a couple of months.  **Postoperatively**, little improvement of symptoms can be expected. However, they often remain. The life expectancy is not long (around 1.5 years), in which they receive radio- and chemotherapy. |
| High-risk endometrium cancer, resection | Resection of high-risk endometrium carcinoma.  **Preoperatively**, patients suffer from vaginal blood loss, weight loss, and weakness.  **Postoperatively**, these patients improve substantially, but the recurrence risk is high. |
| High grade sarcoma, resection | Resection of high grade sarcoma.  **Preoperatively**, these patients have symptoms due to the mass effect of the tumor. The location of the sarcoma impacts the symptoms. For example, the inferior vena cava syndrome is caused by large retroperitoneal masses, or obstipation is caused by subperitoneal masses.These patients often suffer from pain. The surgery is often major, and this impacts preoperative quality of life as well.  **Postoperatively**, these patients recover fully after a successful surgery. However, the recurrence rate is often higher than mild sarcoma (in the extremities). This difference is explained by the technical difficulties associated with the surgery. |
| Instable AP, CABG | Coronary artery bypass graft surgery for instable angina.  **Preoperatively**, these patients have exertion-related symptoms: they have chest pain, and possibly dyspnea. In rest, they are symptom free.  **Postoperatively,** these patients can function as normal. |
| Instable AP, PCI | Percutaneous coronary intervention for instable angina.  **Preoperatively**, these patients have exertion-related symptoms: they have chest pain, and possibly dyspnea. In rest, they are symptom free.  **Postoperatively,** these patients can function as normal. |
| Low-grade glioma, resection | Surgical resection of low-grade glioma.  **Preoperatively,** these patients are often young, where they suffer from epilepsy, or mild neurological deficits. Generally, they have mild symptoms, and can participate in working life. The tumor grows slowly, resulting in reasonable prognosis. They do suffer from knowing that they have a tumor inside their head. There is also considerable suffering from insecurity about recurrence.  **Postoperatively,** these patients have, if a proper resection is able to be performed, a generally good prognosis with reasonable quality of life. Nevertheless, since the treatment is not curative, these patients do suffer from fear for recurrence. Most often, the epilepsy or neurological deficits improve postoperatively. |
| Low-risk endometrium cancer, resection | Resection for low-risk endometrium carcinoma.  **Preoperatively,** these patients have mild vaginal blood loss. Generally, blood loss is the only symptom. These patients can undergo a curative operation with a high survival rate postoperatively. Of course, they do suffer from knowing that they have a potential lethal disease.  **Postoperatively,** they are symptom free, and have a good prognosis. |
| Low grade sarcoma, resection | Surgical resection of a low grade sarcoma.  **Preoperatively,** these patients suffer from a moderately sized sarcoma in their extremities. They can suffer from the knowledge that they have a potentially lethal disease.  **Postoperatively,** these patients are cured, albeit they sometimes require radiotherapy. |
| MI bladder cancer, cystectomy | Cystectomy for muscle-invasive bladder cancer.  **Preoperatively,** these patients first undergo a transurethral resection of their primary tumor (see MI bladder cancer, TUR). However, the pathologist confirms that the tumor is indeed muscle invasive. Therefore, they require a radical cystectomy. This tumor is very aggressive, which impacts the quality of life of patients. These patients also have severe miction problems (hematuria, dysuria).  **Postoperatively**, these patients have a stoma, or neo-bladder, which is connected to the urethra. There is a 50/50 chance on curation. The cured portion of patients often recovers nicely postoperatively, and learn to live with their stoma. |
| MI bladder cancer, TUR | Transurethral resection (TUR) of muscle-invasive bladder cancer.  **Preoperatively,** these patients have non-muscle invasive bladder cancer, which is determined by the pathologist postoperatively. These patients present with hematuria without any other symptoms. Some patients also suffer from miction symptoms, such as frequent urinating, or incontinence. In the TUR procedure, the tumor is removed via the urethra.  **Postoperatively,** these patients are symptom free. Most of them are cured, but they remain in follow up to catch recurrences. |
| Mild salivary gland cancer, resection | Surgical resection of mild salivary gland cancer..  **Preoperatively**, these patients have few symptoms present with an asymptomatic swelling. Patients do suffer from the esthetic of the tumor, and from the knowledge that they have a potentially lethal disease.  **Postoperatively,** these patients can have damaged n. facialis, resulting in hemiparalysis of the face. |
| Ovarium cancer, resection/HIPEC | Hyperthermic intraperitoneal chemotherapy for peritonitis carcinomatosa of ovarium cancer.  **Preoperatively,** these patients have symptoms associated with the large primary tumor, ascites, weight loss, fatigue and weakness.  **Postoperatively,** patients can have a reasonable quality of life, with acceptable symptoms. Nevertheless, the recurrence rate is high. |
| PAD F2, bypass | Peripheral artery bypass surgery for vascular disease.  **Preoperatively**, these patients cannot walk more than 100 meters without claudication complaints.  **Postoperatively**, patients can start with recovery through walking physiotherapy. In most cases the walking distance is extended. Their quality of life will increase, but their vascular disease remains. |
| PAD F3-4, bypass | Peripheral artery bypass surgery for vascular disease.  **Preoperatively**, patients with Fontaine classification 3-4 have restpain and in some cases ischemic ulcers or gangrene.  **Postoperatively**, these patients can move better and will not have any restpain. However, their poor condition and vascular disease remains. |
| Penis cancer, resection | (Partial) penectomy for peniscarcinoma.  **Preoperatively**, patients present with a large, infected, ulceration of the penis, which causes a lot of complaints. Depending on the size of the tumor, a whole or partial penectomy will be performed. During the intervention a sentinel node procedure will be executed as well.  **Postoperatively**, these patients have a good prognosis. After the intervention they will not have any complaints, and still be able to urinate. However, the whole trajectory is uncertain, as their remains a change of recurrence. Besides, the intervention is highly invasive and can cause psychosocial problems. |
| Pit. Adenoma symptomatic, resection | Transphenoidal hypofysectomy for a pituirary gland adenoma. This intervention will only be performed when the adenome produces a high amount of hormones or gives pressure on the optical nerve.  **Preoperatively**, patients with a hormone producing adenoma show symptoms of acromegaly or cushing. Due to the cushing patients can get hypertension, obesitas, diabetes and changes in their behavior.  **Postoperatively**, most patients will be cured. However, they have to take medication for the rest of their lives. When medication is forgotten they can get a Addison’s crisis. |
| Pleurodesis for pneumothorax | Pleurodesis for (recurring), spontaneous (non-traumatic) pneumothorax.  **Preoperatively**, these patients are typically young, tall, smoking, men, who are admitted with dyspnea and chestpain. Primary treatment involves inserting a drain into the thorax, which should cure the pneumothorax. If either a) the drain keeps producing (indicative of a persistent air leak), or b) the pneumothorax recurs up to 3 times, a pleurodesis is performed.  **Postoperatively**, patients are generally cured and asymptomatic. |
| Postrenal obstr, JJ-cath insertion | JJ-catheter insertion for postrenal obstruction. Through the bladder a tube is placed between the kidney and the bladder.  **Preoperatively,**  these patients have a postrenal obstruction due to a tumour or retroperitoneal fibrosis. As patient with malignant or benign diseases are very divergent, only benign causes will be scored. The obstruction causes a hydronephrosis which may reduce kidney function.  **Postoperatively,**  their kidney function will be increased. Every 6 months the JJ-catheter will have to be replaced. They will need the JJ-catheter for the rest of their lives. |
| Rectum carcinoma, resection | Resection of a rectumcarcinoma. Afterwards, they get chemoradiation.  **Preoperatively,** patients have a lot of pain. After intervention 30-50% of the patients will end up with a stoma.  **Postoperatively**, patients rate their quality of life equally to the general population. However, a substantial proportion of patients have a permanent stoma. |
| Renal cancer, partial nefrectomy | Partial nefrectomy for a renal carcinoma.  **Preoperatively,** in most patients the tumor is accidentally discovered. They do not have any complaints. The tumor is approximately 5 cm.  **Postoperatively**, these patients are cured. They often have a quick recovery. |
| Renal cancer, total nefrectomy | Total nefrectomy for a renal carcinoma.  **Preoperatively**, in most patients the tumor is found accidentaly. They present with hematuria. The intervention is curative.  **Postoperatively,** patients have a higher chance of recurrence of disease. When the disease had recurred in most cases metastasis are found as well. At that moment the disease is not curable anymore. |
| Mild salivary gland cancer, resection | Resection of severe salivary gland cancer  **Preoperatively,** patients have no complaints. They usually present with an asymptomatic thickening of the salivary gland. After resection radiotherapy is necessary in most cases.  **Postoperatively,** due to the radiation therapy the facial nerve can be impaired. This occurs in 1-2% of the patients. |
| Severe salivary gland cancer, resection | Resection of severe salivary gland cancer  **Preoperatively,** patients present with a big mass in the head and neck region. This is esthetical difficult. Due to tumor growth the tumor can open the skin. The facial nerve can be infected both by the tumor as by the intervention.  **Postoperatively**, patients will not improve. When the facial nerve is impaired, this cannot be undone. |
| Thyroid adenoma, resection | Partial thyroidectomy for a thyroid adenoma.  **Preoperatively,** patients are often asymptomatic. The tumor is benign and often found by accident. Only in rare cases patients have problems with swallowing of breathing.  **Postoperatively,** patients recover quick and only have a scar in their neck. They do not have to take any medication. |
| Thyroid cancer, resection | Thyroidectomy for a thyroid carcinoma.  **Preoperatively,**  patients are often asymptomatic. They present with a mass in the thyroid. First a partial thyroidectomy is performed. When pathology assessment malignancy confirms as total thyroidectomy is performed. Afterwards patients are treated with radioactive iodine.  **Postoperatively**, patients are cured, but have to take thyroid medication for the rest of their lives. Prognosis of papillary carcinomas is relatively good (90% 10 years survival). In the case of a anaplastic carcinoma (rare) survival is less than a year. |
| UUT cancer, nefroureterectomy | Nefroureterectomy for a ureter carcinoma.  **Preoperatively**, patients present with hematuria. It is the same type of cancer as the bladder carcinoma. It is a curative treatment. Prognosis is comparable to a high grade bladder carcinoma.  **Postoperatively,** patients remain uncertain about their prognosis. The change for metastasis is lower than the high grade bladder carcinoma. |
