# Supplementary material for: Minimizing population health loss due to scarcity in OR capacity: validation of quality of life input
Source: BMC Med Res Methodol. 2023 Jan 31;23:31. doi: 10.1186/s12874-022-01818-z (PMC9887555; doi:10.1186/s12874-022-01818-z)
Supplement: Supplementary file 3 — Additional file 3: Fig. S1. The structure of the previously developed cohort state-transition model. Preop: preoperative state; Postop: postoperative state (6). Fig. S2. The model estimates for urgency based on the original quality of life estimates (upper panel) and the updated scores from both the original and the validation study (bottom panel). Fig. S3. The random effects of procedure on the standard deviation of the QoL estimates. These estimates are the random intercept values for procedure in a model with as independent variable the standard deviations of surgical procedures, also including hospital and pre- or postoperative as fixed effects (supplementary table 2). A random intercept above 0 indicates a higher than expected standard deviation, which we interpret as lower consensus between experts. A random intercept below 0 indicates a lower than expected standard deviation, which we interpret as higher consensus between experts. The overall standard deviation of the random effect was 0.005. Table S1. The estimates from the first mixed effects linear regression model. The dependent variable is the utility scores scored by the expert panel. Table S2. The estimates from the second mixed effects linear regression model. The dependent variable is the standard deviation of the utility scores per study center, pre- and postoperative state, and procedure. Table S3. The quality of life estimates and 95% CI derived from the original study and the validation study, stratified for preoperative and postoperative state, corresponding to figure 1 in the manuscript. Table S4. The difference in urgency of surgical procedures between the original and the updated quality of life estimates. Only the diseases which now include the new scores from the validation study are shown. This table corresponds to figure 4 in the manuscript. [file 12874_2022_1818_MOESM3_ESM.zip › Additional file 3_Fig. S2.docx]

| Original 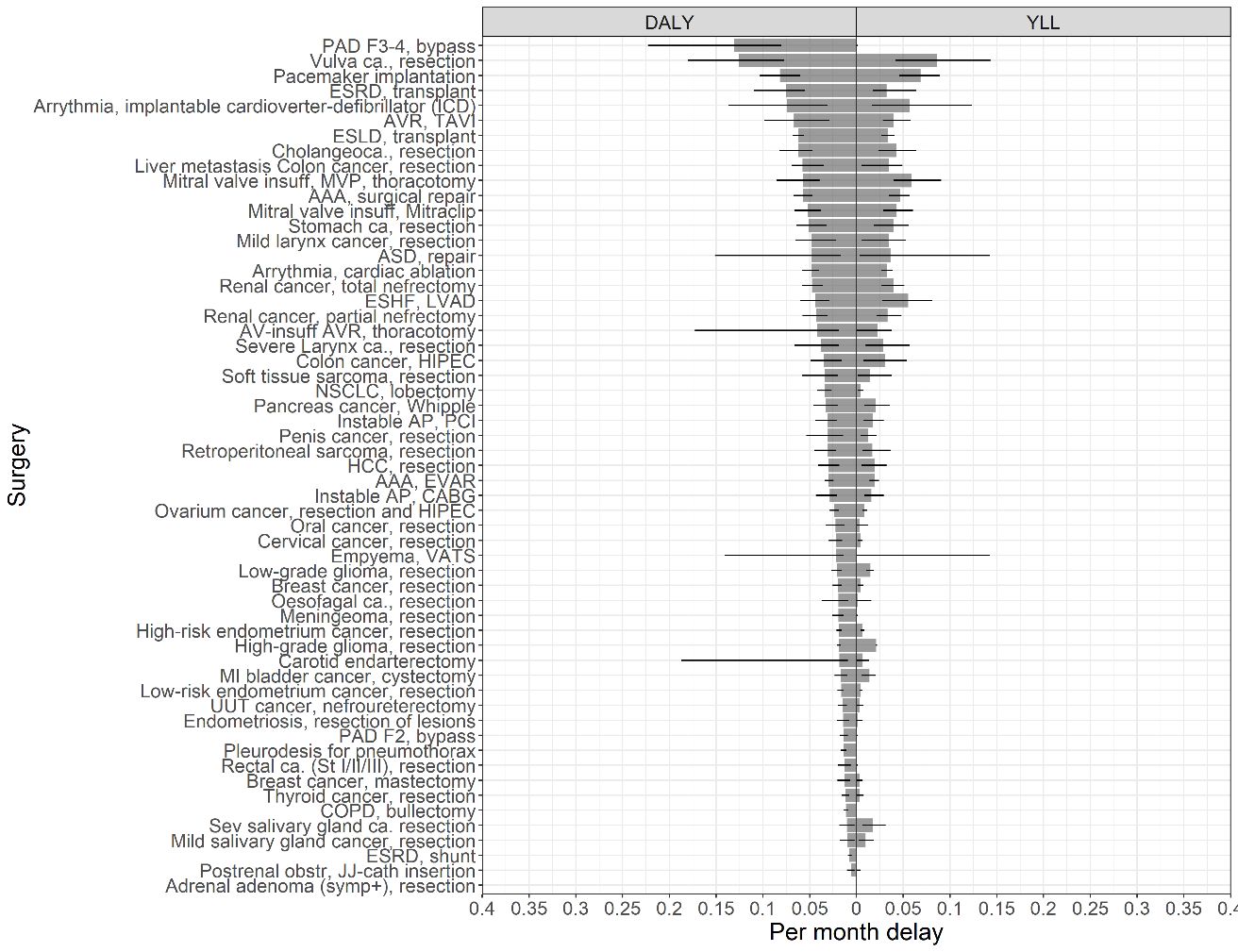 |
| --- |
| Original + validation 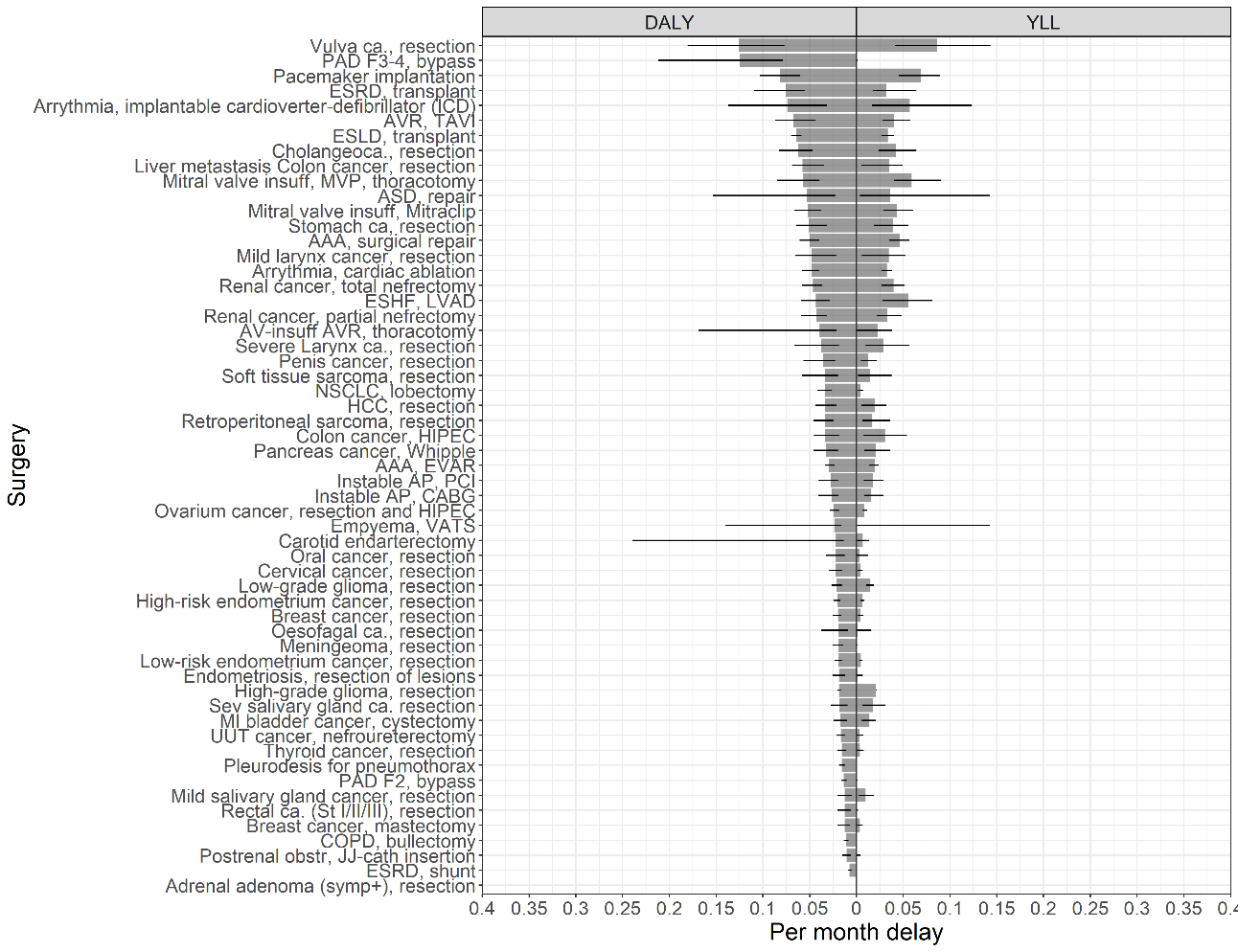 |

Additional file 3: Fig. S2 The model estimates for urgency based on the original quality of life estimates (upper panel) and the updated scores from both the original and the validation study (bottom panel). Abbreviations: AAA, abdominal aneurysm of the aorta; AP, angina pectoris; ASD, atrial septum defect; AV, aortic valve; AVR, aortic valve replacement; CABG, coronary artery bypass graft; COPD, chronic obstructive pulmonary disease; ESHF, end-stage heart failure; ESLD, end-stage liver disease; ESRD, end-stage renal disease; EVAR, endovascular aortic repair; F2, Fontaine 2; F3-4, Fontaine 3-4; HCC, hepatocellular carcinoma; HIPEC, hyperthermic intraperitoneal chemotherapy; LVAD, left ventricle assist device; MI, muscle invasive; NSCLC, non-small cell lung carcinoma; obstr, obstruction; PAD, peripheral arterial disease; PCI, percutaneous coronary intervention; pit, pituitary; sev, severe; TAVI, transcatheter aortic valve implantation; TUR, transurethral resection;UUT, upper urinary tract; VATS, video-assisted thoracoscopy.
